# Electroconvulsive treatment prevents chronic restraint stress‐induced atrophy of the hippocampal formation—A stereological study

**DOI:** 10.1002/brb3.1195

**Published:** 2019-01-18

**Authors:** Johanne L. Alemu, Frederik Elberling, Bushra Azam, Bente Pakkenberg, Mikkel V. Olesen

**Affiliations:** ^1^ Research Laboratory for Stereology and Neuroscience Bispebjerg and Frederiksberg Hospital Copenhagen Denmark; ^2^ Department of Biotechnology and Biomedicine, Disease System Immunology DTU Bioengineering Kongens Lyngby Denmark; ^3^ Institute of Clinical Medicine, Faculty of Health and Medical Sciences University of Copenhagen Copenhagen Denmark

**Keywords:** cell numbers, chronic restraint stress, electroconvulsive stimulation, hippocampal volumes

## Abstract

**Introduction:**

Electroconvulsive therapy (ECT) is one of the most efficient treatments of major depressive disorder (MDD), although the underlying neurobiology remains poorly understood. There is evidence that ECT and MDD exert opposing effects on the hippocampal formation with respect to volume and number of neurons. However, there has been a paucity of quantitative data in experimental models of ECT and MDD.

**Methods:**

Using design‐based stereology, we have measured the effects of a stress‐induced depression model (chronic restraint stress, CRS) and ECS on the morphology of the hippocampus by estimating the volume and total number of neurons in the hilus, CA1, and CA2/3, as well as in the entire hippocampus.

**Results:**

We find that CRS induces a significant decrease in volume exclusively of the hilus and that ECS (CRS + ECS) blocks this reduction. Furthermore, ECS alone does not change the volume or total number of neurons in the entire hippocampus or any hippocampal subdivision in our rat model.

## 1 INTRODUCTION

Major depression (MDD) is a highly debilitating psychiatric disorder with a lifetime prevalence of more than 16% worldwide (World Health Organization, 2017). Although its underlying neurobiology remains elusive, a large body of research implicates dysfunction of the hippocampal formation in the etiology of MDD. The hippocampus has two major anatomic divisions: the hippocampus proper, consisting of three distinct Cornu Ammonis (CA1‐3) regions and strata (oriens, pyramidale, and lacunosum moleculare), and the dentate gyrus, which is comprised of the molecular layer, the granule cell layer, and the hilus, also known as CA4. Previous human postmortem and imaging studies of the hippocampal formation report decreased volumes of the entire hippocampus as well as the dentate gyrus in patients suffering from MDD (Cole, Costafreda, McGuffin, & Fu, 2011; Cole et al., 2010; Gbyl & Videbech, 2018; Huang et al., 2013; McKinnon, Yucel, Nazarov, & MacQueen, 2009; Videbech & Ravnkilde, 2004). Interestingly, these changes are reversed following treatment using antidepressant drugs and electroconvulsive therapy (ECT) (Boldrini et al., 2009, 2013; Huang et al., 2013; Nordanskog et al., 2010), indicating plasticity of the hippocampus. A recent longitudinal MRI study of severely depressed patients showed normalization of hippocampal volume following ECT, an increase sustained for four weeks post‐treatment (Jorgensen et al., 2015). Additionally, a quantitative study of the total number of neurons in the hippocampus showed fewer granule cells in postmortem brain of unmedicated MDD patients compared to patients treated with selective serotonin reuptake inhibitors (Boldrini et al., 2013). Supporting these findings in humans is the extensive literature in rodent models of depression‐like behavior and antidepressant treatment. Accordingly, rodents treated with a range of antidepressant drugs or electroconvulsive stimulation (ECS, the animal model of ECT) show increased hippocampal volume, neurogenesis, angiogenesis, and synaptic differentiation (Chen, Madsen, Wegener, & Nyengaard, 2009, 2010; Hellsten et al., 2005; Kaae, Chen, Wegener, Madsen, & Nyengaard, 2012; Madsen et al., 2000; Olesen, Wörtwein, Folke, & Pakkenberg, 2017; Olesen, Wortwein, & Pakkenberg, 2015; Santarelli et al., 2003). The molecular mechanisms underlying the beneficial effects of antidepressant medication or ECT/ECS are thought to involve alterations in neurotransmitter systems, growth factors, and neurotropic factors (Lee & Son, 2009; Malberg, Eisch, Nestler, & Duman, 2000; Samuels & Hen, 2011; Segi‐Nishida, 2011). In contrast, some previous studies showed “negative” structural effects such as neuronal loss, increased apoptosis, and retraction of dendritic networks in the hippocampus following ECS (Cardoso et al., 2008; Ito et al., 2010; Lukoyanov, Saa, Madeira, & Paula‐Barbosa, 2004). Consequently, it remains uncertain how ECS, in interaction with a depressed state, affects the neuronal population and volume of the hippocampus, and its subregions.

The present study is a direct continuation of two previous stereological studies from our laboratory subjecting rats to three weeks of chronic restraint stress (CRS) and/or a clinical relevant schedule of ECS (Olesen et al., 2017, 2015). Data from these studies showed that CRS induces a depressive‐like phenotype blocked by ECS (CRS + ECS). We also found increased neurogenesis in the subgranular zone (SGZ) that persisted up to 12 months after ECS alone or ECS in conjunction with the CRS model. However, we saw no changes in the volume or the total number of neurons in the granule cell layer following ECS or CRS + ECS, nor did CRS alone influence the hippocampal granule cell layer. In this study, we aimed to quantify the total number of neurons and the volume of the CA1‐3 and hilus as well as the volume of the entire hippocampus using design‐based stereology in the rat CRS + ECS model.

## 2 MATERIALS AND METHODS

### 2.1 Animals and experimental groups

All the animal procedures were performed in accordance with guidelines from the Danish Animal Experimentation Inspectorate and approved by the local ethical committee for experimental animals (License no. 2003/561‐644). Forty‐eight male Wistar rats (Taconic M&B, Denmark), 7–8 weeks old, weighing 220–250 g were used in the experiments. Pairs of rats subjected to the same treatment were housed together in each cage, with a 12‐hr light/dark cycle and ad libitum access to standard rodent chow and tap water. Each treatment group consisted of 12 rats; group one received sham‐ECS (control), group two was subjected to chronic restraint stress (CRS), group three was treated with ECS alone, and group four received a combination of CRS and ECS (CRS + ECS). Before experimentation, the rats acclimatized in their home cage for one week. Three rats died during experimentation. There were no significant differences in the mean body weights between the groups at day 0 of the study (Olesen et al., 2015).

Though not investigated in the present study, the rats were intraperitoneally injected with 100 mg/kg bromodeoxyuridine (5‐bromo‐2‐deoxyuridine [BrdU]; Sigma‐Aldrich, Denmark) dissolved in phosphate‐buffered saline (PBS) once per day (4–5 p.m.) from experimental days 1–21 (Cameron & Mckay, 2001).

### 2.2 Chronic restraint

CRS can provoke depression‐like behavior in rats (Chiba et al., 2012; Watanabe, Gould, & McEwen, 1992). In brief, we administered CRS by confining the rats in a flexible wire mesh restrainer secured in both ends. This limits their freedom to move but allows free breathing and avoids overheating of the animal. In their home cage together with their cage mate, pairs of rats were placed in separate restrainers for 6 hr each day (10 a.m. to 4 p.m.) for 21 consecutive days. During the restraint sessions, the rats were removed from the colony to ensure that rats in the control and ECS groups were not affected by ultrasonic vocalizations from the CRS animals.

### 2.3 Electroconvulsive stimulation

ECS was administered transcranially using a PSCC‐10 pulse stimulator (DCM Electronics, Denmark) as previously described (Olesen et al., 2015), with control animals subjected to the same procedure, but without passage of a current (sham‐ECS). A properly administered ECS causes a generalized tonic–clonic seizure lasting 20–30 s (Larsen et al., 2005). Both sham‐ECS and ECS treatments were given three days per week (Monday, Wednesday, and Friday, 8–9 a.m.) for 3 weeks, approximating the clinical practice of ECT in humans. To minimize postseizure effects, ECS was performed 1 hr prior to the restraint stress sessions. After sham‐ECS or ECS treatments, we returned the rats to their home cage.

### 2.4 Forced swim test

The procedure of the forced swim test was as described previously (Olesen et al., 2015). In brief, on experimental day 22, the rats were individually placed in a Plexiglas cylinder (50 cm in height and 19 cm diameter) containing 28 cm of water (25 ± 0.5°C) and exposed to a 15‐min swim session (pretest). Twenty‐four hours later, the rats were re‐exposed to a five‐min swim session (test). Both sessions were recorded on video, and the total duration of immobility during the test and the final 5 min of the pretest were recorded. As sham‐ECS, CRS, and ECS treatments preceded the behavioral testing, we calculate the difference in % immobility between the test and pretest to correct for variability of the behavioral responsiveness in individual rats (Hageman, Nielsen, Wortwein, Diemer, & Jorgensen, 2009). The rats were considered immobile when floating in an upright position and making only small movements to keep their heads above water (Stogner & Holmes, 2000).

### 2.5 Tissue processing

Twenty‐four hours after the behavioral assessment, the rats were anesthetized using hypnorm/midazolam and then transcranially perfused using PBS followed by 4% paraformaldehyde (PFA). The brains were then carefully removed from the skull, weighed, and postfixated overnight in 4% PFA. The following day brains were transferred to a 30% sucrose (w/v) solution in 0.1 M phosphate buffer for three days at 4°C. Finally, the brains were frozen on dry ice and kept at −80°C until sectioning. Prior to sectioning, the two hemispheres were separated, and one was chosen randomly (with the following hemispheres chosen systematically left/right) before being mounted on an ULTRApro 5000 Cryostat (Bright Instruments, UK). Then, the complete hippocampus was sectioned (Bregma −1.80 to −7.04; Paxinos & Watson, 1986) into 80‐µm‐thick coronal sections, and finally, every 5th section was subsampled with a random start between sections one and five for final cell counting and structure volume estimation.

### 2.6 Tissue staining

The sections were mounted on SuperFrost + slides and allowed to air‐dry for 40 min at room temperature. Then, the slides were rehydrated in dH_2_O for 10 min, followed by staining in 0.02% cresyl violet (#C5042, Sigma‐Aldrich, Switzerland) for 15 min. This process was repeated before the slides were dehydrated in 96% ethanol for 5 min and 99% for 2 × 2 min. Finally, slides were placed in xylene for 2 × 15 min before being coverslipped using Pertex mounting medium (#00801, Histolab, Sweden). Note: The sections were BrdU immunostained prior to cresyl violet staining, results of which we report in our previous study of neurogenesis (Olesen et al., 2015).

### 2.7 Stereology

**Figure 1:**
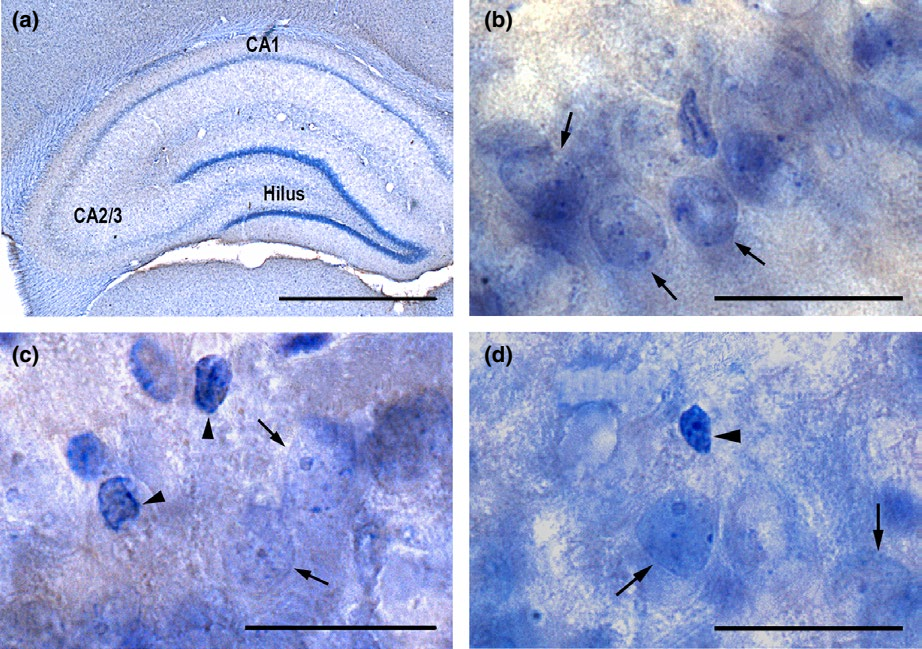
Representative histological image showing the dorsal hippocampus at ×1.25 magnification (a). Panels (b–d) show additional magnification (×60) of the CA1 (B), CA2/3 (c), and hilus (d). Arrows indicate neurons, and arrowheads indicate glial cells. Scale bars = 2,500 μm (a) and 50 μm (b–d)

We estimated the total number of neurons in the pyramidal cell layer of the CA1 and CA2/3 as well as the total number of neurons in the hilus using the fractionator design and optical disectors (Gundersen, 1986; West, Slomianka, & Gundersen, 1991). In brief, the fractionator design states that the total number of neurons (*N*) is estimated by multiplying the total number of cells counted (∑*Q* ^‐^) with the reciprocal sampling fractions:

$$N=\frac{1}{\mathrm{ssf}}*\frac{1}{\mathrm{asf}}*\frac{{\overset{¯}{t}}_{Q^{-}}}{h}*\sum Q^{-}$$

$${\overset{¯}{t}}_{Q^{-}}=\frac{\sum_{i}\left(t_{i}q_{i}^{-}\right)}{\sum_{i}q_{i}^{-}}$$

where *ssf* is the section sampling fraction, *asf* is the area sampling fraction, ${\overset{¯}{t}}_{Q^{-}}$ is the number‐weighted mean section thickness, *h* is the height of the disector, and *q_i_* is the number of neurons counted per section (Dorph‐Petersen, Nyengaard, & Gundersen, 2001; Fabricius, Wörtwein, & Pakkenberg, 2008). The cell counts were completed using the new‐CAST software (ver. 5.3.0.1464, Visiopharm, Hørsholm, Denmark) and obtained using a microscope (Olympus DP72) equipped with a 100× oil immersion objective (numerical aperture = 1.40) and a motorized stage. Unbiased rectangular counting frames (CA1: 420 µm^2^; CA2/3:700 µm^2^; hilus: 3,800 µm^2^) were superimposed on the tissue magnified on a computer screen (final onscreen magnification = 3,000×), and movements in the *z*‐direction were measured with a digital microcator (Heidenhain, VRZ 401). The counting was performed using a disector height of 10 µm, and guard zones of 5 µm at the top of the section and 10–15 µm at the bottom. Additionally, uniform distribution of neurons in the disector height was confirmed by analyzing the *z*‐distribution. The shrinkage was assessed by measuring the height of each section. The average thickness of the sections, originally cut to 80 µm, was 27.9–29.3 µm (for stereological sampling scheme, see Table 1). We used morphological criteria to identify the neurons, which are characterized by a clearly defined nucleus with colorless cytoplasm and dark nucleolus (for representative micrographs, see Figure 1). The same sections were used to determine the volumes of the CA1 and CA2/3 pyramidal cell layer, the hilus, and the entire hippocampus, all of which were estimated using Cavalieri's estimator (Gundersen & Jensen, 1987). Finally, the unilateral cell numbers and volumes were multiplied by two to obtain the total bilateral cell numbers and volumes. The sub‐regions used for cell counting and volume estimation were based on delineations found in the literature (Paxinos & Watson, 1986; West et al., 1991).

**Table 1:** Sampling scheme for estimation of neuron numbers and volumes

	Control	CRS	ECS	CRS + ECS
Number of sections				
CA1	8–11	8–11	9–12	8–12
CA2/3	8–11	8–12	9–12	8–12
Hilus	10–12	9–12	9–12	8–13
Total hippocampus	11–13	11–14	11–14	10–13
Section thickness (μm)				
CA1	29.1 ± 1.4	28.0 ± 1.1	28.6 ± 1.3	28.1 ± 0.9
CA2/3	28.8 ± 1.4	28.7 ± 1.1	29.3 ± 1.3	28.1 ± 1.1
Hilus	27.9 ± 1.2	28.6 ± 1.1	29.0 ± 1.5	29.1 ± 0.9
Total hippocampus	80	80	80	80
Number of counting frames				
CA1	64 (55–79)	72 (60–89)	65 (51–91)	72 (55–83)
CA2/3	64 (52–79)	67 (54–100)	64 (48–87)	65 (52–79)
Hilus	75 (60–89)	67 (55–86)	75 (54–91)	74 (58–89)
Total hippocampus	221(201–265)	226 (205–278)	230 (202–269)	235 (208–276)
Number of neurons sampled				
CA1	159 (115–215)	177 (127–247)	154 (108–198)	179 (131–245)
CA2/3	124 (101–200)	140 (91–178)	122 (91–147)	127 (85–172)
Hilus	205 (132–286)	245 (122–348)	204 (131–299)	237 (165–317)

*Note.* Only volume estimations were performed in total hippocampus. Section thickness is represented as mean ± standard error of mean (*SEM*) in parentheses, whereas the number of counting frames and the number of sampled neurons are represented as mean with range in parentheses.

CRS: chronic restraint stress; ECS: electroconvulsive stimulation.

### 2.8 Statistics

One‐way ANOVA followed by Tukey's multiple comparisons test was used to analyze the data. Shapiro–Wilk normality test was used to examine the normal distribution and the Brown–Forsythe test together with Bartlett's test to test for equal variance between groups. Values exceeding mean ± 2 × *SD* were considered as outliers. The coefficient of variance (CV) and the coefficient of error (CE, Gundersen, Jensen, Kiêu, & Nielsen, 1999) were calculated to estimate the precision of the estimates and are shown in Table 2. The statistics and graphical presentation were performed using GraphPad Prism 7 (version 7.02, GraphPad Software Inc., La Jolla, CA, USA), and data are presented as dot plots with horizontal bars indicating the mean. The level of significance was set to *p* < 0.05.

**Table 2:** Numerical estimation of the total number of neurons and volumes

	Control	CE	CRS	CE	ECS	CE	CRS + ECS	CE
Volume (mm^3^)								
CA1	2.49 (0.13)	0.05	2.80 (0.15)	0.05	2.42 (0.13)	0.06	2.86 (0.09)‡	0.05
CA2/3	3.18 (0.12)	0.06	3.21 (0.12)	0.05	3.10 (0.11)	0.06	3.26 (0.12)	0.05
Hilus	4.31 (0.15)	0.07	3.63 (0.09)†	0.05	4.51 (0.13)	0.06	4.17 (0.17)	0.05
Entire hippocampus	58.6 (0.07)	0.02	59.7 (0.06)	0.02	61.1 (0.06)	0.02	62.3 (0.06)	0.02
Neurons (10^3^)								
CA1	532 (0.15)	0.08	561 (0.12)	0.08	502 (0.14)	0.08	573 (0.17)	0.08
CA2/3	299 (0.16)	0.09	354 (0.15)	0.09	322 (0.14)	0.09	317 (0.28)	0.09
Hilus	108 (0.22)	0.07	101 (0.14)	0.06	100 (0.24)	0.08	115 (0.29)	0.07

*Note.* Please note that entire hippocampal volumes do not apply to the sum of the subregions. CV values are represented in parenthesis.

CE: coefficient of error; CRS: chronic restraint stress; ECS: electroconvulsive stimulation.

^†^ *p* < 0.05 versus control, ^‡^ *p* < 0.05 versus ECS (*n* = 10–12 per group).

## 3 RESULTS

### 3.1 Hippocampal volumes

**Figure 2:**
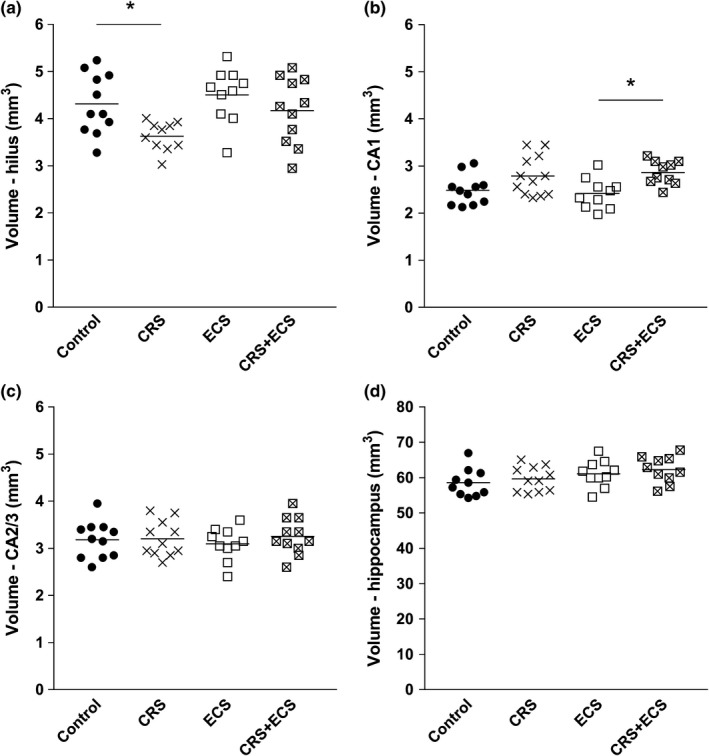
Quantitative volumetric data (mm^3^) of the rat hippocampal formation including the hilus (a), CA1 (b), CA2/3 (c), and entire hippocampus (d). Horizontal bars represent mean values. Abbreviations: CRS, chronic restraint stress; ECS, electroconvulsive stimulation. *p < 0.05 (*n* = 10–12 per group)

Table 2 and Figure 2 present the stereological estimates of hippocampal volumes. One‐way ANOVA demonstrated a significant volumetric effect of treatments in the hilus (*F*_3,38_ = 4.214, *p* = 0.011) and CA1 (*F*_3,39_ = 4.516, *p* = 0.008). When compared to the control group, Tukey's multiple comparisons test showed a significant 16% decrease in the volume of the hilus in the CRS alone group (*p* = 0.049, Figure 2a), whereas the volume of the CA1 was statistically significantly increased by 18% in rats treated with CRS + ECS compared to ECS alone (*p* = 0.026, Figure 2b). We saw no volumetric alterations following ECS alone, nor did we find any changes of the volume of the CA2/3 (*F*_3,39_ = 0.322, *p* = 0.809, Figure 2c) or the entire hippocampus in the ECS and CRS treatment groups (*F*_3,37_ = 1.903, *p* = 0.146, Figure 2d).

### 3.2 Total number of neurons

**Figure 3:**
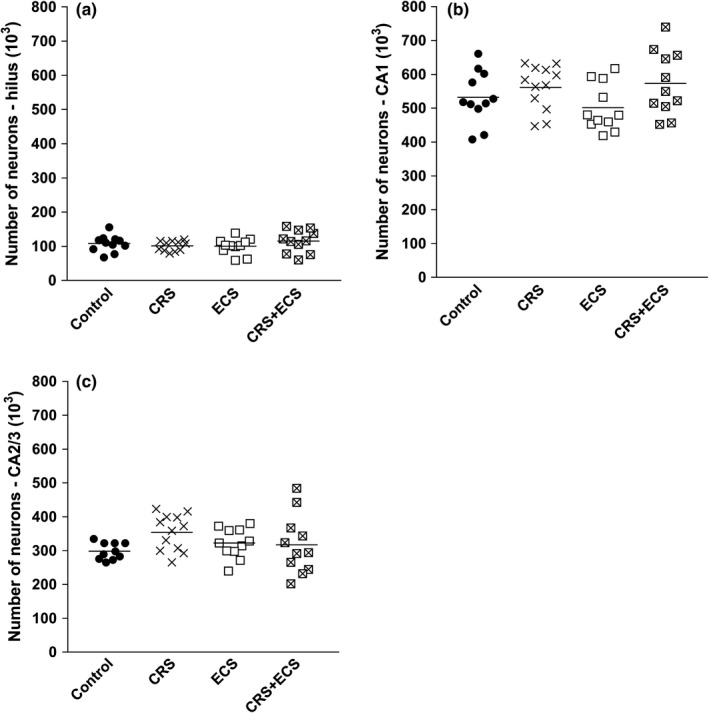
Quantitative data of the total number of neurons (10^3^) in the hilus (a), CA1 (b), and CA2/3 (c) of the rat hippocampus. Horizontal bars represent mean values. Abbreviations: CRS, chronic restraint stress; ECS, electroconvulsive stimulation (*n* = 10–12 per group)

Table 2 and Figure 3 show the stereological estimates of the total number of neurons in hippocampal subregions. Our results show no significant effects of CRS, ECS, or CRS + ECS on the total number of neurons in the hilus (*F*_3,40_ = 0.872, *p* = 0.464, Figure 3a), CA1 (*F*_3,41_ = 1.902, *p* = 0.114, Figure 3b), or CA2/3 (*F*_3,40_ = 1.788, *p* = 0.165, Figure 3c).

## 4 DISCUSSION

The primary finding of the present stereological study is a region‐specific decrease in the volume of the hippocampal hilus in depression model rats, which normalized to control level in the group also receiving ECS. This CRS‐induced hilar shrinkage did not result in a loss of neurons or other alterations in the entire hippocampus or its subregions. Furthermore, we demonstrate that ECS and CRS + ECS in combination do not significantly affect the volume or the total number of neurons resident in the CA1, CA2/3, hilus, or the complete hippocampus as compared to the control group.

In the exact same rats as used in this study, we have previously shown that CRS significantly increases depression‐like behavior in the FST (Percent immobility: Control = 6.3%, CRS = 14.2%, *p* < 0.05) and that the elevated immobility is normalized following ECS (Percent immobility: CRS + ECS = 7.7%; Olesen et al., 2015). Our present estimations of volumes in the hippocampal formation show significantly lower volume of the dentate hilus of the CRS group. This result is partly in line with recent studies displaying shrinkage of the dentate gyrus following unpredictable restraint stress in rats as well as in untreated MDD patients (Huang et al., 2013; Schoenfeld, McCausland, Morris, Padmanaban, & Cameron, 2017). On the other hand, CRS did not cause changes in the volume of the CA1, CA2/3, or the entire hippocampus. These results stand in contrast to several previous human and rodent studies, which demonstrated decreased volume of the complete hippocampus, (including CA regions) in MDD patients (Campbell & MacQueen, 2004; Cole et al., 2011, 2010; Huang et al., 2013; Jorgensen et al., 2015; McKinnon et al., 2009; Nordanskog et al., 2010; Schmaal et al., 2016; Videbech & Ravnkilde, 2004). Comparable results are reported in a number of studies in animal models of depression‐like behavior (Kaae et al., 2012; Lee, Jarome, Li, Kim, & Helmstetter, 2009; Luo et al., 2014; Schoenfeld et al., 2017). We suppose that these discrepant results may be related to the use of different delineations of the CA regions. Previous studies have shown that shrinkage of the CA3 is due to dendritic atrophy of pyramidal neurons (Hageman, Nielsen, Wortwein, Diemer, & Jorgensen, 2008; Heine, Maslam, Zareno, Joels, & Lucassen, 2004; Schoenfeld et al., 2017). Since we defined the CA regions at the level of pyramidal cell bodies, our procedure may have missed potential dendritic volumetric changes in the CA1 and CA2/3 in the present study. Conversely, the disagreement in total volumes of the entire hippocampus between this study and previous semiquantitative reports cannot be explained alone by dendritic atrophy or diverging delineations. We included the subiculum in the results presented herein, but neither was there any changes in the mean total volume of the hippocampus defined without the subiculum (Control = 53.35 mm^3^ [range: 49.95–60.21 mm^3^], ECS = 55.05 mm^3^ [range: 50.22–58.86 mm^3^], CRS = 53.87 mm^3^ [range: 50.49–59.40 mm^3^], CRS + ECS = 56.53 mm^3^ [range: 49.41–62.37 mm^3^]; *p* = 0.140). Moreover, some previous stereological and MRI studies lend support to the present findings by showing no changes in hippocampal volume in depressed patients (Cao et al., 2017; Cobb et al., 2013). Although we are unable to exclude the occurrence of possible changes in dendritic plasticity of the hippocampal CA1 and CA2/3, our data indicate that any such changes or decreases in the hilar volume must have minor impact on our estimations of the total hippocampal volume.

Interestingly, application of ECS to depression model rats (CRS + ECS) had no significant effect on the volume of the hilus compared to that in controls. This is in line with a previous stereological study demonstrating reduced hippocampal volume in a genetic rat model of depression, which normalized following chronic ECS (Kaae et al., 2012). Also, MRI studies and meta‐analyses of severely depressed patients show a decreased volume of the hippocampus as well as hippocampal subdivisions, changes that are not observed in patients receiving antidepressant medication (Huang et al., 2013; Malykhin, Carter, Seres, & Coupland, 2010; Videbech & Ravnkilde, 2004) or ECT (Gbyl & Videbech, 2018; Jorgensen et al., 2015; Nordanskog et al., 2010). Consequently, our results suggest that ECS can normalize a reduced hilar volume possibly by altering various neurotransmitter and hormone systems; for example, glucocorticoid‐induced changes in processes which are thought to be involved in major depression (Pariante & Miller, 2001), effects that are opposed using antidepressant drugs (Calfa et al., 2003; Raone et al., 2007), and ECT (Kunugi et al., 2006; Yuuki et al., 2005). In general, this normalization may also apply to other paradigms of rodent depression models and likewise in case of ECT treatment of patients suffering from MDD. This conjecture is further supported by our result of unaltered volume of the hippocampus or its subdivisions following ECS alone. However, a few previous quantitative studies have reported that ECS alone can *increase* the volume of the entire hippocampus, GCL, and hilus in naïve rats (Chen et al., 2009; Kaae et al., 2012). Whereas our study used a clinically relevant schedule of ECS (three times per week for three weeks), animals in the other studies received ECS for ten consecutive days. Thus, the observed volumetric discrepancies may at least in part be explained by differing experimental conditions. This speculation is further supported by a study by Lukoyanov et al. (2004) showing no changes following five consecutive days of ECS. To our surprise, we observe a difference in the mean volume of the CA1 between ECS‐ and ECS + CRS‐treated rat groups. However, neither volume differed significantly from the control result, suggesting that the effect may have arisen from individual group differences rather than treatment effects.

The present study shows no significant changes in the total number of neurons after CRS, ECS, and CRS + ECS in any hippocampal regions examined. Similarly, some previous stereological studies report no significant changes in the total number of neurons in the rat CA1 and CA2/3 following chronic imipramine (Chen, Madsen, Wegener, & Nyengaard, 2008) or ECS treatment (Lukoyanov et al., 2004). However, the latter study did report fewer in the hilus in rats with six consecutive ECS sessions, a result that we cannot confirm or reject due to our different experimental design. Even though our ECS procedure differs from several former studies using acute and repeated ECS administration (Ito et al., 2010; Kaae et al., 2012; Malberg et al., 2000), we consider that our design better emulates the clinical practice.

Three weeks of daily CRS did not alter the total number of neurons in any hippocampal subdivision, which stands in contrast to a previous study showing increased apoptosis in the CA3 (Sapolsky, 2000). The authors of that study hypothesized that stress would increase apoptosis in CA2/3 due to increased glucocorticoid or CRS levels. In accordance with that hypothesis, a more recent semiquantitative study of CRS (six hours each day for 10 consecutive days) reported fewer neurons throughout the hippocampal CA regions (CA1‐3), which was indeed associated with increased apoptosis (Orlovsky, Dosenko, Spiga, Skibo, & Lightman, 2014). Although we did not measure apoptosis in the present study, the methods for cell quantitation present a parsimonious explanation for the differing results. Our present use of stereological cell counting provides unbiased estimates approximating the true value. Thus, we find that any stress‐induced increase in apoptosis seems to have been insufficient in significantly reducing the total number of neurons. Hence, molecular mechanisms other than apoptosis and loss of neurons may play a crucial role in CRS‐induced depression‐like behavior.

Limitations to the present study include our focus on the number of neurons and volume of the hippocampus and its anatomic divisions in relation to the CRS‐induced depression model. Semiquantitative studies in humans show diverging findings for volumetric changes in the hippocampus of patients with first major depressive episode (MDE) compared to those with recurrent depressive episodes (Cobb et al., 2013; Cole et al., 2011, 2010; McKinnon et al., 2009; Schmaal et al., 2016). The CRS paradigm used in our study better emulates first MDE than recurrent episodes, which may partly describe our finding of unaltered hippocampal volume. We suppose that subjecting animals to “blocks” of repeated CRS might better model the induction of recurrent depressive episodes and thus improve our understanding of the link between hippocampal changes and MDD pathophysiology.

In conclusion, this paper shows that CRS causes a decrease in the volume of the hippocampal hilus which is normalized to control levels after ECS treatment (CRS + ECS). The hilar atrophy does not result in a changed number of neurons or other modifications of the entire hippocampus or its subregions. Finally, ECS and CRS + ECS have no effects on the total number of neurons and volume in the CA1, CA2/3 and hilus, or the volume of the entire hippocampus. In the context of our previous findings in the exact same animals (Olesen et al., 2015, 2017), our compiled results show that CRS induces depression‐like behavior and hilar atrophy in rats, effects that are rescued following a clinical relevant schedule of ECS. Furthermore, CRS do not affect basal neurogenesis, the number of preexisting neurons in any hippocampal regions examined, or the volume of granule cell layer, CA1, CA2/3, or the entire hippocampus. Finally, ECS alone causes a significant and long‐lasting (up to one year) increase in the formation of new neurons in the subgranular zone/granule cell layer but has no effects on the number of pre‐existing neurons or the volume of the entire hippocampus or its subdivisions. Using mediation analysis, we also show that ECS‐induced neurogenesis per se cannot account for the antidepressant effects of ECS in this model, implying that molecular mechanism other than apoptosis and neuronal loss may mediate CRS‐induced depression‐like behavior.

## CONFLICT OF INTEREST

Nothing to declare.

## References

[brb31195-bib-0001] Boldrini, M. , Underwood, M. D. , Hen, R. , Rosoklija, G. B. , Dwork, A. J. , Mann, J. J. , & Arango, V. (2009). Antidepressants increase neural progenitor cells in the human hippocampus. Neuropsychopharmacology, 34, 2376–2389. 10.1038/npp.2009.75 19606083 PMC2743790

[brb31195-bib-0002] Boldrini, M. , Santiago, A. N. , Hen, R. , Dwork, A. J. , Rosoklija, G. B. , Tamir, H. , … Mann, J. (2013). Hippocampal granule neuron number and dentate gyrus volume in antidepressant‐treated and untreated major depression. Neuropsychopharmacology, 38, 1068–1077. 10.1038/npp.2013.5 23303074 PMC3629406

[brb31195-bib-0003] Calfa, G. , Kademian, S. , Ceschin, D. , Vega, G. , Rabinovich, G. A. , & Volosin, M. (2003). Characterization and functional significance of glucocorticoid receptors in patients with major depression: Modulation by antidepressant treatment. Psychoneuroendocrinology, 28, 687–701. 10.1016/S0306-4530(02)00051-3 12727135

[brb31195-bib-0004] Cameron, H. A. , & McKay, R. D. (2001). Adult neurogenesis produces a large pool of new granule cells in the dentate gyrus. Journal of Comparative Neurology, 435, 406–417. 10.1002/cne.1040 11406822

[brb31195-bib-0005] Campbell, S. , & MacQueen, G. (2004). The role of the hippocampus in the pathophysiology of major depression. Journal of Psychiatry & Neuroscience, 29, 417–426. 15644983 PMC524959

[brb31195-bib-0006] Cao, B. , Passos, I. C. , Mwangi, B. , Amaral‐Silva, H. , Tannous, J. , Wu, M. J. , … Soares, J. C. (2017). Hippocampal subfield volumes in mood disorders. Molecular Psychiatry, 22, 1352–1358. 10.1038/mp.2016.262 28115740 PMC5524625

[brb31195-bib-0007] Cardoso, A. , Assuncao, M. , Andrade, J. P. , Pereira, P. A. , Madeira, M. D. , Paula‐Barbosa, M. M. , & Lukoyanov, N. V. (2008). Loss of synapses in the entorhinal‐dentate gyrus pathway following repeated induction of electroshock seizures in the rat. Journal of Neuroscience Research, 86, 71–83. 10.1002/jnr.21474 17705293

[brb31195-bib-0008] Chen, F. , Madsen, T. M. , Wegener, G. , & Nyengaard, J. R. (2008). Changes in rat hippocampal CA1 synapses following imipramine treatment. Hippocampus, 18, 631–639. 10.1002/hipo.20423 18306301

[brb31195-bib-0009] Chen, F. , Madsen, T. M. , Wegener, G. , & Nyengaard, J. R. (2009). Repeated electroconvulsive seizures increase the total number of synapses in adult mate rat hippocampus. European Neuropsychopharmacology, 19, 329–338. 19176277 10.1016/j.euroneuro.2008.12.007

[brb31195-bib-0010] Chen, F. , Madsen, T. M. , Wegener, G. , & Nyengaard, J. R. (2010). Imipramine treatment increases the number of hippocampal synapses and neurons in a genetic animal model of depression. Hippocampus, 20, 1376–1384. 10.1002/hipo.20718 19921703

[brb31195-bib-0011] Chiba, S. , Numakawa, T. , Ninomiya, M. , Richards, M. C. , Wakabayashi, C. , & Kunugi, H. (2012). Chronic restraint stress causes anxiety‐ and depression‐like behaviors, downregulates glucocorticoid receptor expression, and attenuates glutamate release induced by brain‐derived neurotrophic factor in the prefrontal cortex. Progress in Neuro‐psychopharmacology & Biological Psychiatry, 39, 112–119. 10.1016/j.pnpbp.2012.05.018 22664354

[brb31195-bib-0012] Cobb, J. A. , Simpson, J. , Mahajan, G. J. , Overholser, J. C. , Jurjus, G. J. , Dieter, L. , … Stockmeier, G. A. (2013). Hippocampal volume and total cell numbers in major depressive disorder. Journal of Psychiatric Research, 47, 299–306. 10.1016/j.jpsychires.2012.10.020 23201228 PMC3757567

[brb31195-bib-0013] Cole, J. , Costafreda, S. G. , McGuffin, P. , & Fu, C. H. Y. (2011). Hippocampal atrophy in first episode depression: A meta‐analysis of magnetic resonance imaging studies. Journal of Affective Disorders, 134, 483–487. 10.1016/j.jad.2011.05.057 21745692

[brb31195-bib-0014] Cole, J. , Toga, A. W. , Hojatkashani, C. , Thompson, P. , Costafreda, S. G. , Cleare, A. J. , … Fu, C. H. Y. (2010). Subregional hippocampal deformations in major depressive disorder. Journal of Affective Disorders, 126, 272–277. 10.1016/j.jad.2010.03.004 20392498 PMC3197834

[brb31195-bib-0015] Dorph‐Petersen, K. A. , Nyengaard, J. R. , & Gundersen, H. J. G. (2001). Tissue shrinkage and unbiased stereological estimation of particle number and size. Journal of Microscopy, 204, 232–246. 10.1046/j.1365-2818.2001.00958.x 11903800

[brb31195-bib-0016] Fabricius, K. , Wörtwein, G. , & Pakkenberg, B. (2008). The impact of maternal separation on adult mouse behaviour and on the total neuron number in the mouse hippocampus. Brain Structure and Function, 212, 403–416. 10.1007/s00429-007-0169-6 18200448 PMC2226080

[brb31195-bib-0017] Gbyl, K. , & Videbech, P. (2018). Electroconvulsive therapy increases brain volume in major depression: a systematic review and meta‐analysis. Acta Psychiatrica Scandinavica, 138, 180‐195. 10.1111/acps.12884 29707778

[brb31195-bib-0018] Gundersen, H. J. G. (1986). Stereology of arbitrary particles – A review of unbiased number and size estimators and the presentation of some new ones, in memory of Thompson, William, R. Journal of Microscopy, 143, 3–45. 10.1111/j.1365-2818.1986.tb02764.x 3761363

[brb31195-bib-0019] Gundersen, H. J. G. , & Jensen, E. B. (1987). The efficiency of systematic sampling in stereology and its prediction. Journal of Microscopy, 147, 229–263. 10.1111/j.1365-2818.1987.tb02837.x 3430576

[brb31195-bib-0020] Gundersen, H. J. G. , Jensen, E. B. , Kiêu, K. , & Nielsen, J. (1999). The efficiency of systematic sampling in stereology – Reconsidered. Journal of Microscopy, 19, 199–211. 10.1046/j.1365-2818.1999.00457.x 10348656

[brb31195-bib-0021] Hageman, I. , Nielsen, M. , Wortwein, G. , Diemer, N. H. , & Jorgensen, M. B. (2008). Electroconvulsive stimulations prevent stress‐induced morphological changes in the hippocampus. Stress, 11, 282–289. 10.1080/10253890701783794 18574788

[brb31195-bib-0022] Hageman, I. , Nielsen, M. , Wortwein, G. , Diemer, N. H. , & Jorgensen, M. B. (2009). Electroconvulsive stimulations normalizes stress‐induced changes in the glucocorticoid receptor and behavior. Behavioral Brain Research, 196, 71–77. 10.1016/j.bbr.2008.07.037 18725247

[brb31195-bib-0023] Heine, V. M. , Maslam, S. , Zareno, J. , Joels, M. , & Lucassen, P. J. (2004). Suppressed proliferation and apoptotic changes in the rat dentate gyrus after acute and chronic stress are reversible. European Journal of Neuroscience, 19, 131–144. 10.1046/j.1460-9568.2003.03100.x 14750971

[brb31195-bib-0024] Hellsten, J. , West, M. J. , Arvidsson, A. , Ekstrand, J. , Jansson, L. , Wennstrom, M. , & Tingstrom, A. (2005). Electroconvulsive seizures induce angiogenesis in adult rat hippocampus. Biological Psychiatry, 58, 871–878. 10.1016/j.biopsych.2005.05.023 16043138

[brb31195-bib-0025] Huang, Y. S. , Coupland, N. J. , Lebel, R. M. , Carter, R. , Seres, P. , Wilman, A. H. , & Malykhin, N. V. (2013). Structural changes in hippocampal subfields in major depressive disorder: A high‐field magnetic resonance imaging study. Biological Psychiatry, 74, 62–68. 10.1016/j.biopsych.2013.01.005 23419546

[brb31195-bib-0026] Ito, M. , Seki, T. , Liu, J. A. , Nakamura, K. , Namba, T. , Matsubara, Y. , … Arai, H. (2010). Effects of repeated electroconvulsive seizure on cell proliferation in the rat hippocampus. Synapse (New York, N.Y.), 64, 814–821. 10.1002/syn.20796 20340175

[brb31195-bib-0027] Jorgensen, A. , Magnusson, P. , Hanson, L. G. , Kirkegaard, T. , Benveniste, H. , Lee, H. , … Jorgensen, M. B. (2015). Regional brain volumes, diffusivity, and metabolite changes after electroconvulsive therapy for severe depression. Acta Psychiatrica Scandinavica, 133, 154–164. 10.1111/acps.12462 26138003

[brb31195-bib-0028] Kaae, S. S. , Chen, F. H. , Wegener, G. , Madsen, T. M. , & Nyengaard, J. R. (2012). Quantitative hippocampal structural changes following electroconvulsive seizure treatment in a rat model of depression. Synapse (New York, N. Y.), 66, 667–676. 10.1002/syn.21553 22389166

[brb31195-bib-0029] Kunugi, H. , Ida, I. , Owashi, T. , Kimura, M. , Inoue, Y. , Nakagawa, S. , … Mikuni, M. (2006). Assessment of the dexamethasone/CRH test as a state‐dependent marker for hypothalamic‐pituitary‐adrenal (HPA) axis abnormalities in major depressive episode: A Multicenter Study. Neuropsychopharmacology, 31, 212–220. 10.1038/sj.npp.1300868 16123748

[brb31195-bib-0030] Larsen, M. H. , Olesen, M. , Woldbye, D. P. D. , Hay‐Schmidt, A. , Hansen, H. H. , Ron, L. C. B. , & Mikkelsen, J. D. (2005). Regulation of activity‐regulated cytoskeleton protein (Arc) mRNA after acute and chronic electroconvulsive stimulation in the rat. Brain Research, 1064, 161–165. 10.1016/j.brainres.2005.09.039 16309632

[brb31195-bib-0031] Lee, E. , & Son, H. (2009). Adult Hippocampal neurogenesis and related neurotrophic factors. BMB Reports, 42, 239–244. 10.5483/BMBRep.2009.42.5.239 19470236

[brb31195-bib-0032] Lee, T. , Jarome, T. , Li, S. J. , Kim, J. J. , & Helmstetter, F. J. (2009). Chronic stress selectively reduces hippocampal volume in rats: A longitudinal magnetic resonance imaging study. NeuroReport, 20, 1554–1558. 10.1097/WNR.0b013e328332bb09 19858767 PMC2783199

[brb31195-bib-0033] Lukoyanov, N. V. , Saa, M. J. , Madeira, M. D. , & Paula‐Barbosa, M. M. (2004). Selective loss of hilar neurons and impairment of initial learning in rats after repeated administration of electroconvulsive shock seizures. Experimental Brain Research, 154, 192–200. 10.1007/s00221-003-1658-3 14557909

[brb31195-bib-0034] Luo, Y. , Cao, Z. , Wang, D. , Wu, L. , Li, Y. , Sun, W. , & Zhu, Y. (2014). Dynamic study of the hippocampal volume by structural MRI in a rat model of depression. Neurological Sciences, 35, 1777–1783. 10.1007/s10072-014-1837-y 24929958

[brb31195-bib-0035] Madsen, T. M. , Treschow, A. , Bengzon, J. , Bolwig, T. G. , Lindvall, O. , & Tingstrom, A. (2000). Increased neurogenesis in a model of electroconvulsive therapy. Biological Psychiatry, 47, 1043–1049. 10.1016/S0006-3223(00)00228-6 10862803

[brb31195-bib-0036] Malberg, J. E. , Eisch, A. J. , Nestler, E. J. , & Duman, R. S. (2000). Chronic antidepressant treatment increases neurogenesis in adult rat hippocampus. Journal of Neuroscience, 20, 9104–9110. 10.1523/JNEUROSCI.20-24-09104.2000 11124987 PMC6773038

[brb31195-bib-0037] Malykhin, N. V. , Carter, R. , Seres, P. , & Coupland, N. J. (2010). Structural changes in the hippocampus in major depressive disorder: Contributions of disease and treatment. Journal of Psychiatry & Neuroscience, 35, 337–343. 10.1503/jpn.100002 20731966 PMC2928287

[brb31195-bib-0038] McKinnon, M. C. , Yucel, K. , Nazarov, A. , & MacQueen, G. M. (2009). A meta‐analysis examining clinical predictors of hippocampal volume in patients with major depressive disorder. Journal of Psychiatry & Neuroscience, 34, 41–54. 19125212 PMC2612082

[brb31195-bib-0039] Nordanskog, P. , Dahlstrand, U. , Larsson, M. R. , Larsson, E. M. , Knutsson, L. , & Johanson, A. (2010). Increase in hippocampal volume after electroconvulsive therapy in patients with depression a volumetric magnetic resonance imaging study. Journal of ECT, 26, 62–67. 10.1097/YCT.0b013e3181a95da8 20190603

[brb31195-bib-0040] Olesen, M. V. , Wörtwein, G. , Folke, J. , & Pakkenberg, B. (2017). Electroconvulsive stimulation results in long‐term survival of newly generated hippocampal neurons in rats. Hippocampus, 27, 52–60. 10.1002/hipo.22670 27756104

[brb31195-bib-0041] Olesen, M. V. , Wortwein, G. , & Pakkenberg, B. (2015). Electroconvulsive stimulation, but not chronic restraint stress, causes structural alterations in adult rat hippocampus‐a stereological study. Hippocampus, 25, 72–80. 10.1002/hipo.22351 25139647

[brb31195-bib-0042] Orlovsky, M. A. , Dosenko, V. E. , Spiga, F. , Skibo, G. G. , & Lightman, S. L. (2014). Hippocampus remodeling by chronic stress accompanied by GR, proteasome and caspase‐3 overexpression. Brain Research, 1593, 83–94. 10.1016/j.brainres.2014.09.059 25285893

[brb31195-bib-0043] Pariante, C. M. , & Miller, A. H. (2001). Glucocorticoid receptors in major depression: Relevance to pathophysiology and treatment. Biological Psychiatry, 49, 391–404. 10.1016/S0006-3223(00)01088-X 11274650

[brb31195-bib-0044] Paxinos, G. , & Watson, C. (1986). The rat brain—In stereotaxic coordinates. San Diego, CA: Academic Press.

[brb31195-bib-0045] Raone, A. , Cassanelli, A. , Scheggi, S. , Rauggi, R. , Danielli, B. , & De Montis, M. G. (2007). Hypothalamus‐pituitary‐adrenal modifications consequent to chronic stress exposure in an experimental model of depression in rats. Neuroscience, 146, 1734–1742. 10.1016/j.neuroscience.2007.03.027 17481824

[brb31195-bib-0046] Samuels, B. A. , & Hen, R. (2011). Neurogenesis and affective disorders. European Journal of Neuroscience, 33, 1152–1159. 10.1111/j.1460-9568.2011.07614.x 21395859

[brb31195-bib-0047] Santarelli, L. , Saxe, M. , Gross, C. , Surget, A. , Battaglia, F. , Dulawa, S. , … Hen, R. (2003). Requirement of hippocampal neurogenesis for the behavioral effects of antidepressants. Science, 301, 805–809. 10.1126/science.1083328 12907793

[brb31195-bib-0048] Sapolsky, R. M. (2000). Glucocorticoids and hippocampal atrophy in neuropsychiatric disorders. Archives of General Psychiatry, 57, 925–935. 10.1001/archpsyc.57.10.925 11015810

[brb31195-bib-0049] Schmaal, L. , Veltman, D. J. , van Erp, T. G. , Sämann, P. G. , Frodl, T. , Jahanshad, N. , … Hibar, D. P. (2016). Subcortical brain alterations in major depressive disorder: Findings from the ENIGMA Major Depressive Disorder working group. Molecular Psychiatry, 21, 806–812. 10.1038/mp.2015.69 26122586 PMC4879183

[brb31195-bib-0050] Schoenfeld, T. J. , McCausland, H. C. , Morris, H. D. , Padmanaban, V. , & Cameron, H. A. (2017). Stress and loss of adult neurogenesis differentially reduce hippocampal volume. Biological Psychiatry, 82, 914–923. 10.1016/j.biopsych.2017.05.013 28629541 PMC5683934

[brb31195-bib-0051] Segi‐Nishida, E. (2011). Exploration of new molecular mechanisms for antidepressant actions of electroconvulsive seizure. Biological and Pharmaceutical Bulletin, 34, 939–944. 10.1248/bpb.34.939 21719995

[brb31195-bib-0052] Stogner, K. A. , & Holmes, P. V. (2000). Neuropeptide‐Y exerts antidepressant‐like effects in the forced swim test in rats. European Journal of Pharmacology, 387, R9–R10. 10.1016/S0014-2999(99)00800-6 10650166

[brb31195-bib-0053] Videbech, P. , & Ravnkilde, B. (2004). Hippocampal volume and depression: A meta‐analysis of MRI studies. American Journal of Psychiatry, 161, 1957–1966. 10.1176/appi.ajp.161.11.1957 15514393

[brb31195-bib-0054] Watanabe, Y. , Gould, E. , & McEwen, B. S. (1992). Stress induces atrophy of apical dendrites of hippocampal CA3 pyramidal neurons. Brain Research, 588, 341–345. 10.1016/0006-8993(92)91597-8 1393587

[brb31195-bib-0055] West, M. J. , Slomianka, L. , & Gundersen, H. J. G. (1991). Unbiased stereological estimation of the total number of neurons in the subdivisions of the rat hippocampus using the optical fractionator. The Anatomical Record, 231, 482–497. 10.1002/ar.1092310411 1793176

[brb31195-bib-0056] World Health Organization (WHO) . (2017). Depression: let’s talk. Retrieved from https://www.who.int/mental_health/management/depression/en/.

[brb31195-bib-0057] Yuuki, N. , Ida, I. , Oshima, A. , Kumano, H. , Takahashi, K. , Fukuda, M. , … Mikuni, M. (2005). HPA axis normalization, estimated by DEX/CRH test, but less alteration on cerebral glucose metabolism in depressed patients receiving ECT after medication treatment failures. Acta Psychiatrica Scandinavica, 112, 257–265. 10.1111/j.1600-0447.2005.00625.x 16156832

